# Impact of colchicine on mortality and morbidity in COVID-19: a systematic review

**DOI:** 10.1080/07853890.2021.1993327

**Published:** 2022-03-08

**Authors:** Devang Sanghavi, Pankaj Bansal, Ikwinder Preet Kaur, Mohsin Sheraz Mughal, Chandana Keshavamurthy, Austin Cusick, Jennifer Schram, Siva Naga S. Yarrarapu, Abhishek R. Giri, Nirmaljot Kaur, Pablo Moreno Franco, Andy Abril, Fawad Aslam

**Affiliations:** a Department of Critical Care Medicine, Mayo Clinic – Florida, Jacksonville, FL, USA; b Department of Rheumatology, Mayo Clinic Health System, Eau Claire, WI, USA; c Department of Internal Medicine, Rutgers/Monmouth Medical Center, Long Branch, NJ, USA; d Department of Rheumatology, Ochsner Medical Center, New Orleans, LA, USA; e Department of Internal Medicine, Riverside Methodist Hospital, Columbus, OH, USA; f Mayo Clinic Libraries – Wisconsin, Mayo Clinic Health System, Eau Claire, WI, USA; g Department of Internal Medicine, RWJ Barnabas Health, Monmouth Medical Center, Long Branch, NJ, USA; h Department of Internal Medicine, Riverside School of Medicine, University of California, Riverside, CA, USA; i Department of Rheumatology, Mayo Clinic – Florida, Jacksonville, FL, USA; j Department of Rheumatology, Mayo Clinic – Arizona, Scottsdale, AZ, USA

**Keywords:** Colchicine, COVID-19, Coronavirus

## Abstract

**Introduction:**

Colchicine, because of its anti-inflammatory and possible anti-viral properties, has been proposed as potential therapeutic option for COVID-19. The role of colchicine to mitigate “cytokine storm” and to decrease the severity and mortality associated with COVID-19 has been evaluated in many studies.

**Objective:**

To evaluate the role of colchicine on morbidity and mortality in COVID-19 patients.

**Methods:**

This systematic review was conducted in accordance with the PRISMA recommendations. The literature search was conducted in 6 medical databases from inception to February 17, 2021 to identify studies evaluating colchicine as a therapeutic agent in COVID-19. All included studies were evaluated for risk of bias (ROB) using the Revised Cochrane ROB tool for randomised controlled trials (RCTs) and Newcastle-Ottawa Scale (NOS) for case-control and cohort studies.

**Results:**

Four RCTs and four observational studies were included in the final analysis. One study evaluated colchicine in outpatients, while all others evaluated inpatient use of colchicine. There was significant variability in treatment protocols for colchicine and standard of care in all studies. A statistically significant decrease in all-cause mortality was observed in three observational studies. The risk of mechanical ventilation was significantly reduced only in one observational study. Length of hospitalisation was significantly reduced in two RCTs. Risk for hospitalisation was not significantly decreased in the study evaluating colchicine in outpatients. Very few studies had low risk of bias.

**Conclusion:**

Based on the available data, colchicine shall not be recommended to treat COVID-19. Further high-quality and multi-center RCTs are required to assess the meaningful impact of this drug in COVID-19.

**KEY MESSAGES**
- Colchicine, an anti-inflammatory agent has demonstrated anti-viral properties in in-vitro studies by degrading the microtubules, as well as by inhibiting the production of pro-inflammatory cytokines.
- Colchicine has been studied as a potential therapeutic option for COVID-19, with variable results.
- Until further research can establish the efficacy of colchicine in COVID-19, the use of colchicine in COVID-19 shall be restricted to clinical trials.

## 1. Introduction

Emanating from the Wuhan region of China in 2019, the coronavirus disease (COVID-19) has since spread and caused a global catastrophe. As of 13th July 2021, the disease caused by the Severe Acute Respiratory Syndrome Coronavirus (SARS-CoV-2) is responsible for more than 4 million deaths across the world [1]. The treatment options are limited with only one Food and Drug Authority-approved drug (remdesivir). Some medicines have received emergency use authorisation, while there are ongoing trials on several other agents that have shown plausible efficacy in preliminary studies [2–7]. Understandably, there is a great unmet need for therapeutic options. The pathogenesis of moderate to severe COVID-19 is centred around the “cytokine storm”, where the rapid upsurge in inflammatory cytokines is responsible for the multiple-organ failure and increased severity of the disease [8]. Therefore, anti-inflammatory agents like dexamethasone and tocilizumab have shown promise [2]. The known anti-inflammatory properties of colchicine are utilised in several disorders including but not limited to gout, Familial Mediterranean fever (FMF), acute and recurrent pericarditis, Behcet disease, Sweet syndrome, and calcium pyrophosphate deposition disease [9]. Colchicine, an alkaloid isolated from colchicum autumnale plant (molecular formula C22H25NO6), binds to the intracellular unpolymerised protein tubulin irreversibly, forming a tubulin-colchicine complex, which prevents polymerisation of the microtubule polymer, hence arresting the microtubule growth and promoting microtubule depolymerisation. Colchicine has potential synergy in the treatment of cytokine cascade in COVID-19 at different levels. *In vitro* studies demonstrate the functionality of microtubules during initial cellular infection with SARS-CoV-2. By degrading the microtubules, colchicine is hypothesised to exude antiviral properties. The coronavirus spike protein utilises the cytoskeletal elements of host cells during viral entry. Colchicine hence may hamper viral entry, infection, and propagation due to the multi-faceted uses of microtubule elements. A component of the SARS-associated coronavirus called viroporin-E creates calcium-permeable ion channels and activates the NLRP3 inflammasome. Colchicine disrupts the NLRP3 inflammasome activation, which plays an important role in the development of phase 3 cytokine storm from SARS-COV 2. Furthermore, colchicine may interfere with the cytokine storm by inhibiting the production of pro-inflammatory cytokines such as IL-1β, IL-18, IL-6, and IFN- γ and superoxide free radicals [8–11]. There have been several studies investigating the role of colchicine in the management of COVID-19. These have shown variable results. The objective of this study was to systematically review the available literature on the role of colchicine in the treatment of COVID-19.

## 2. Materials and methods

### 2.1. Eligibility criteria

This systematic review was conducted in accordance with the PRISMA (Preferred Reporting Items for Systematic Reviews and Meta-Analysis) recommendations [12].

#### 2.1.1. Principle for study design (PICOS)

Population: Adults (18 years and older) with COVID-19.

Intervention: Use of colchicine for COVID-19.

Comparator/Control: Other treatment modalities including standard of care as documented in the studies.

Outcomes: All-cause mortality, mechanical ventilation requirement, risk of hospitalisation, length of hospital stay, effect on inflammatory markers (C-reactive protein, ferritin, D-dimer, lactate dehydrogenase or any other inflammatory markers as mentioned in the studies), and adverse effects (gastrointestinal upset or any other adverse effects as reported in the studies).

Study design: Randomised controlled trials (RCTs), prospective and retrospective cohort studies.

#### 2.1.2. Inclusion and exclusion criteria

Studies evaluating the use of colchicine for treatment of COVID-19 in adults were included for this systematic review. Exclusion criteria included the use of colchicine beyond COVID-19, animal studies, case reports, case series, review articles, meta-analyses, non-English language studies, and those without a comparator arm.

### 2.2. Search strategy and sources for information

An experienced medical librarian developed and conducted the literature search in OVID EMBASE, PubMed, medRxiv, Scopus, Prospero, and Google Scholar. All databases were searched from database inception to February 17, 2021. The searches were limited to the English language. MeSH and keywords search terms included: “covid”, “covid-19”, “SARS-CoV-2”, “Coronavirinae”, “severe acute respiratory syndrome coronavirus 2”, “sars cov 2”, “ncov”, “2019 ncov”, “colchicine”, “Colcrys”, and “mitigare”. The details of the search strategy are provided in the Appendix 1. Bibliographies of identified studies and abstracts published in the annual conferences of professional medicine subspecialties including rheumatology, critical care, infectious disease, internal medicine, and emergency medicine were searched to identify additional studies.

### 2.3. Study selection and data collection

We used EndNote version 20.1 for data management and citation duplication assessment. Two authors independently reviewed the identified abstracts to identify articles for full-text review. The full text was also reviewed if the abstract was unavailable. Reasons for exclusion were recorded. A third author independently reviewed the results from both authors and resolved any conflicts. Relevant information from the included papers was extracted by two authors and re-examined for accuracy by a third author. Pertinent data extracted included study first author, publication date, study location, study design, study participants number and baseline characteristics, study interventions, and study outcomes.

### 2.4. Assessment of methodologic quality

The risk of bias for RCTs was assessed using the Revised Cochrane risk-of-bias tool for randomised trials [13]. For retrospective observational studies and cohort studies, the Newcastle-Ottawa Scale (NOS) for case-control and cohort studies was used, respectively [14]. For the NOS, a score of 6 or more was considered to be suggestive of higher study quality and study credibility [15]. One author assessed the risk of bias in the included studies, and the results were reviewed by other authors. Disagreements were resolved by group discussion and consensus. The signalling questions and quality assessment definitions are given in Appendix 2.

## 3. Results

### 3.1. Review of literature/study characteristics

**Figure 1.**
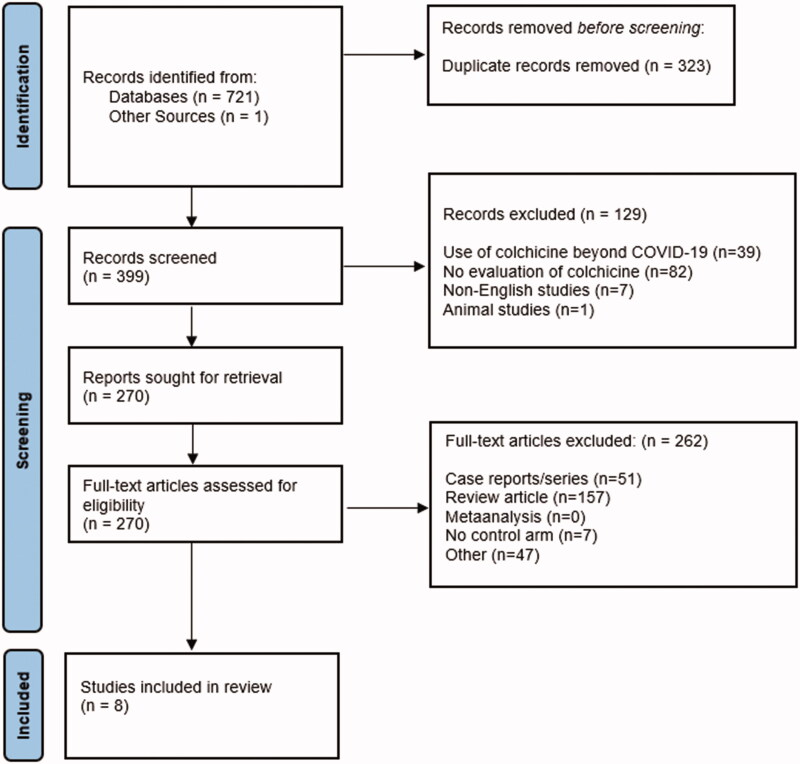
PRISMA flow diagram.

The initial database search identified a total of 721 citations. A total of eight studies containing 5661 patients were included in the final analysis [16–23]. A PRISMA flow diagram describing the inclusion process is mentioned in Figure 1. Of the included studies, 4 were randomised controlled trials (RCTs) and 4 were observational studies. In the RCTs, 2 were double-blinded, 1 was open-label and 1 was single-blinded & open-label design. In the observational studies, 3 were retrospective and 1 was a prospective study. Two studies were published as preprints. One RCT was performed in Canada and recruited patients from six countries, 2 studies were conducted in the US, 1 in India, 1 in Brazil, 1 in Iran, 1 in Greece, and 1 in Italy. Only one RCT included outpatients, all other studies were performed on inpatients. The COLCORONA trial by Tardif et al. was terminated early at 75% of enrolment due to logistical and time constraints [16]. The GRECCO trial by Deftereos et al. was terminated early due to slow patient enrolment [17].

### 3.2. Patient characteristics

Seven studies restricted inclusion to RT-PCR confirmed cases, while one recruited clinically suspected COVID-19 patients. Baseline patient characteristics in the colchicine and control groups were separately mentioned by 7 studies (Table 1). The mean age of patients in the colchicine and control group in these seven studies was 61 and 61.7 years respectively. The colchicine group contained 46.8% of males and the control group had 51.0% males. Only two studies reported race – In the study by Tardif et al., there were 93.3% Caucasians in the colchicine group and 93.2% Caucasians in the control group [16]. In the study by Burnetti et al., the colchicine group had 26.8% white and 48.8% Hispanics, while the control group had 26.0% whites and 48.8% Hispanics [23]. Information about patient baseline comorbidities in the studies is detailed in Table 1.

**Table 1.** Study characteristics and outcomes.

	Salehzadeh et al. [19]	Lopes et al. [18]	Tardif et al. [16]	Mahale et al. [20]	Scarsi et al. [21]	Sandhu et al [22]	Deftereos et al. [17]	Brunetti et [23]
study design	RCT, open label, and single-blinded	RCT, double-blinded, placebo controlled	RCT, double-blind, placebo controlled	Retrospective cohort	Retrospective case-control	Prospective case-control	RCT, open label	Retrospective propensity score matched cohort
Study setting	Inpatient	Inpatient	Outpatient	Inpatient	Inpatient	Inpatient	Inpatient	Inpatient
Participants	Colchicine + HCQ vs Placebo + HCQ	Colchicine + SoC vs Placebo + SoC	Colchicine vs Placebo	HCQ + MP + colchicine vs SoC	Colchicine + SoC vs Placebo + SoC	Colchicine + SoC vs SoC	Colchicine + SoC vs SoC	Colchicine + SoC vs SoC
Use of steroids in SoC	No	Yes	No	Yes	Yes	Yes	No	No
Study timings	21 May–20 June 2020	11 April–31 August 2020	March 2020–January 2021	22 March– 31 May 2020	19 March– 5 April 2020	March 21, 2020–May 02, 2020	3 April–27 April 2020	1 March 2020–30 May 2020
No. of participants (colchicine vs comparator group)	50 vs 50	36 vs 36	2235 vs 2253	39 vs 95	122 vs 140	53 vs 144	55 vs 50	41 vs 262
Age (years)	56.56 vs 55.56 (median)	54.5 vs 55.0 (median)	54.4 vs 54.9 (mean)	NA	69.3 vs 70.5 (mean)	67.7 vs 66.4 (mean)	63 vs 65 (median)	61.2 vs 63.0 (mean)
Male gender (%)	38 vs 44	53 vs 39	44.6 vs 47.5	NA	63 vs 64	61.8 vs 51.3	56.4 vs 60.0	68.3 vs 70.7
Comorbidities								
Hypertension (%)	6 vs 16	NA	34.9 vs 37.6	NA	NA	52.9 vs 71.8	40.0 vs 50.0	51.2 vs 53.1 After propensity matching-60.6 vs 36.4, *p* .049
Diabetes (%)	10 vs 12	36 vs 42	19.9 vs 20.0	NA	NA	32.4 vs 51.3	16.4 vs 24.0	19.5 vs 32.1, *p* .047 Not statistically significant after propensity matching
Respiratory disease (%)	0 vs 8	11 vs 14	26.1 vs 26.9	NA	17 vs 22	17.4 vs 17.7	5.5 vs 4.0	17.1 vs 11.5
Coronary artery disease (%)	12 vs 18	47 vs 33	2.9 vs 3.2	NA	NA	5.9 vs 7.7	16.4 vs 10.0	12.2 vs 8.8
Others					Malignancies: 9% vs 21%, *p* value .013	Renal failure: 35.2% vs 65.3%, *p* .015		
Colchicine dose and duration	1mg daily for 6 days	0.5 mg thrice daily for 5 days followed by 0.5 mg twice daily for 5 days.	0.5 mg twice daily for 3 days followed by once daily for 27 Days.	0.5 mg daily for 1 week	1 mg daily	0.6 mg twice daily for 3 days followed by 0.6 mg once daily for 12 days or discharge.	1.5 mg followed by 0.5 mg 60 min later on day 1, then 0.5 mg twice daily till discharge or 21 days.	Loading Dose 1.2 mg on day 1 followed by 0.6 mg twice daily.
Timing of colchicine initiation	Timing from symptom onset to enrolment 6.28 days colchicine vs 8.12 days Placebo	NA	Within 4 h of enrolment in the study	Within 5 days of symptoms onset in 70% of patients	Within a mean of 1 day of hospitalisation in most cases (% not mentioned)	NA	Time from hospital admission to enrolment 3 days colchicine, 5 days placebo	Within 72 h of hospitalisation in 69.7% (*n* = 23) of patients
Outcomes								
All-cause mortality (%)	0 vs 0	0 vs 5.6	0.2 vs 0.4; p 0.08	28.2 vs 26.3	16.3 vs 37.1; *p* < .001	47.1 vs 80.8; *p* .0003	1.8 vs 8.0	9.1 vs 33.3; *p* .023
Inflammatory markers (CRP, LDH, D-dimer, Ferritin) difference	NA	Colchicine group had a significant reduction in LDH and CRP	NA	NA	NA	Significant lower %age delta values of D-dimer, CRP, and ferritin in colchicine group	Peak D-dimer was significantly lower in Colchicine group	Colchicine group had a significant reduction in CRP
Mechanical ventilation (%)	NA	NA	0.5 vs 0.9	38.5 vs 26.3	NA	47.1 vs 87.2; *p* < .0001	1.8 vs 10.0	2.44 vs 0.76
Length of hospital stay (days)	6.3 vs 8.1	6.0 vs 8.5	NA	NA	NA	10.5 vs 11.0	12 vs 13	NA

RCT: Randomised Controlled Trial; HCQ: hydroxychloroquine; SoC: standard of care; MP: methylprednisolone; CRP: C-reactive protein; LDH: lactate dehydrogenase; NA: not available.

### 3.3. Treatment data

Standard of care (SOC- protocol to treat COVID-19 patients) was highly variable depending on physician discretion, drug availability, and institutional protocol. This included hydroxychloroquine (HCQ), azithromycin, ceftriaxone, antivirals, IL-6 inhibitor, anticoagulation, and corticosteroids in various combinations. There was no standardised approach on dosing and duration of colchicine. It was variable in each study as mentioned in Table 2. The dose was adjusted according to the weight of the patient, GFR, other treatments, and side effects severity. In the largest study by Tardif et al evaluating outpatient use of colchicine in COVID-19, colchicine was initiated within 4 h of enrolment [16]. In the studies evaluating inpatient colchicine use, the interval between hospital admission and colchicine administration was variable and ranged from within 72 h to 6.28 days after hospitalisation.

**Table 2.** Study criteria.

Study	Inclusion criteria	Exclusion criteria	Primary outcomes	Secondary outcomes
Salehzadeh et al. [19]	Lung CT-scan compatible with COVID-19 and a positive COVID-19 RT-PCR	Sensitivity to any medications of regimens, renal failure, heart failure, pregnancy, participation in another clinical study and refusal to participate in the study before or during the follow-up period	Duration of hospitalisation, symptoms and coexistent diseases	Mortality and morbidity, re-admission and symptoms (examined 2 weeks after discharge)
Lopes et al. [18]	Moderate or severe COVID-19 diagnosed by RT-PCR and lung CT scan compatible with COVID-19, patients older than 18 years, body weigh*t* > 50 kg, QT interval <450 ms	Mild COVID-19 or in need for ICU admission, diarrhoea resulting in dehydration, abnormal calcium and potassium levels, known allergy to colchicine, porphyria, myasthenia gravis or uncontrolled arrhythmia, pregnancy or lactation, metastatic cancer or immunosuppressive chemotherapy, regular use of digoxin, amiodarone, verapamil or protease inhibitors; chronic liver disease with hepatic failure	The need for supplemental oxygen, duration of hospitalisation, ICU admission, duration of ICU stay, mortality rate	CRP, LDH, neutrophil/lymphocyte ratio at Days 0 and 7, adverse events, QTc prolongation >450 ms
Tardif et al. [16]	Patients 40 years or older, diagnosed with COVID-19 [PCR confirmed or clinical diagnosis] within 24 h of enrolment, and at least one of the following high-risk criteria: age of 70 years or older, BMI 30 kg/m2 or more, diabetes, uncontrolled hypertension, respiratory disease, heart failure, coronary artery disease, fever of at least 38.4 °C within the last 48 h, dyspnoea at the time of presentation, bicytopenia, pancytopenia, or the combination of high neutrophil and low lymphocyte counts	IBD, chronic diarrhoea or malabsorption, neuromuscular disease, eGFR < 30, severe liver disease, current treatment with colchicine, current chemotherapy, significant sensitivity to colchicine	Composite of death or hospitalisation due to COVID-19 by day 30	Composites of primary and need for mechanical ventilation by day 30
Mahale et al. [20]	RT-PCR-positive COVID-19 patients aged more than 18 years requiring oxygen therapy within 72 h of their hospital admission	Patients who were already on steroids or immunosuppressant drugs, imminent death within 24 h of hospital admission (more than two organ failures on admission)	In-hospital mortality, need for mechanical ventilation, discharge from hospital	Duration of hospital and ICU stay
Scarsi et al. [21]	Virologically and radiographically confirmed COVID-19 patients	eGFR < 30 mL/min	Survival rate at 21 days	Clinical and laboratory variables associated with survival
Sandhu et al. [22]	RT-PCR confirmed COVID-19. Patients who had at least two separate time-point measurements for at least two out of four serum inflammatory markers (CRP, D-dimer, ferritin, or LDH) were selected for the final comprehensive analysis	Pregnancy, end-stage renal disease, concurrent use of protease inhibitor, ketoconazole, cyclosporine, clarithromycin, lamivudine, dolutegravir, tocilizumab or convalescent plasma	Duration of hospitalisation, all-cause mortality, need for mechanical ventilation, discharge rate from the hospital	Change in serum ferritin, CRP, LDH and D-dimer
Deftereos et al. [17]	RT-PCR confirmed cases with a temperature of 37.5 °C or greater and 2 or more of the following: sustained coughing, sustained sore throat, anosmia and/or ageusia, fatigue and/or tiredness, and arterial oxygen partial pressure lower than 95 mmHg on room air	Hepatic failure, eGFR < 20 mL/min/1.73m^2^, QTc >/=450 ms, need of early mechanical ventilation, pregnancy or lactation, hypersensitivity to colchicine	Maximum HS-cardiac troponin level, time for CRP to reach more than 3× upper limit normal, clinical deterioration by 2 points on a 7-grade clinical status scale	Need for mechanical ventilation, all-cause mortality, adverse events
Brunetti et al. [23]	RT-PCR confirmed cases	Not mentioned	All-cause in-hospital mortality within the 28-day follow-up	Favorable change in OSCI on days 14 and 28 versus baseline, the proportion of patients with a WHO OSCI score of < 4 (indicating proportion of patients not requiring supplemental oxygen on days 14 and 28, and proportion of patients discharged by day 28)

RT-PCR: Reverse transcriptase-polymerase chain reaction; eGFR: estimated glomerular filtration rate; OSCI: modified Ordinal Scale for Clinical Improvement; WHO: World Health Organisation; HS: high sensitivity; QTc: corrected; QT: interval; ms: milliseconds.

### 3.4. Outcomes

#### 3.4.1. All-cause mortality

All studies assessed all-cause mortality. A statistically significant difference was observed in 3 studies. Sandhu et al reported a significant decrease in mortality in the colchicine + SOC group (47.1% vs 80.8%: *p*-value .0003) [22]. The follow-up duration is unclear. At a 28 days follow-up period, Burnetti et al reported a significant reduction in mortality after propensity matching (9.1% vs 33.3%; *p*-value .023) [23]. However, the results were not significant before matching (9.8% vs 22.1%; *p* = .077). Colchicine was associated with a significant reduction in mortality after adjustment for age, comorbidity index, and c-reactive protein (odds ratio, 0.21; 95% confidence interval, 0.06–0.71; *p* = .012). Similarly, Scarsi et al reported a significant decrease in mortality in the colchicine + SOC as compared to the placebo + SOC group (16.3% vs 37.1%; *p* < .001) at 21 days follow-up [21]. On performing a cox proportional hazards regression survival analysis, a lower risk of death was independently associated with colchicine treatment (HR = 0.151 (95% CI 0.062–0.368), *p* < .0001). There was no death observed in both groups in the study by Salezadeh et al. [19] In the study by Tardif et al, results were not significant at 30 days follow-up period (mean value) [16]. Deftereos et al observed a decrease in death rate in the colchicine group (1.8% vs 8.0%; *p*-value not mentioned) [17]. None of the patients recruited to the colchicine group died in the study by Lopes et al (0.0 vs 5.6; *p*-value not mentioned) and Mahale et al (28.2 vs 26.3; *p*-value not mentioned) observed higher mortality in the colchicine group [18,20]. Event-free survival (duration from the primary clinical endpoints) was reported by Deftereos et al. [17]. The mean event-free survival time was increased in the colchicine group (20.7 days vs 18.6 days: *p*-value .03).

#### 3.4.2. Inflammatory markers

The effects of an intervention on inflammatory markers (CRP, Lactate Dehydrogenase, Ferritin, D-dimer) was reported in 50% of included studies. Lopes et al. reported that both groups had similar levels of serum CRP at day 0 followed by a significant reduction in CRP in the colchicine group compared to the baseline CRP and compared to the placebo group (*p* < .001) at day 4 [18]. The values became near the normal range (median = 1.3 mg/dL) for the colchicine group on day 4. For the placebo group, the statistical difference compared with the baseline occurred at day 7 (*p* < .001), but no return to a normal range of median CRP was observed in controls. Similarly, the post-test for LDH showed a difference between day zero and days 4 and 7 for the colchicine group. Deftereos et al. reported that the peak d-dimer was lower (*p* = .04) in the colchicine group and CRP was not statistically significant between colchicine + SOC vs SOC [17]. Sandhu et al. reported a significant decrease in percentage delta values of D-dimer (P 0.037), CRP (P 0.014), and ferritin (*p* .012) levels in colchicine + SOC vs SOC [22]. Brunetti et al. reported that a repeat CRP after colchicine administration (available only for 36%) showed a significant reduction in mean CRP from baseline (14.8 vs 7.8 mg/dL; *p* = .021) [23].

#### 3.4.3. Mechanical ventilation and need for oxygen

Only one study reported a significant decrease in the need for mechanical ventilation in the colchicine + SOC group; 47.1% vs 87.2%; *p* < .0001 [22]. The results were not statistically significant in 1 study (0.5% vs 1% odds ratio, 0.50; 95% CI, 0.23–1.07) [16]. Lopes et al. did not analyse patients requiring mechanical ventilation [18]. Statistical significance was not reported in 3 studies (Deftereos et al 1.8% vs 10%, Brunetti et al 2.4% vs 0.7%, Mahale et al 38.5% vs 26.3%) [17,20,23]. The need for mechanical ventilation was not reported in 2 studies [19,21]. The study performed by Salehzadeh et al was only performed in non-ICU patients and did not evaluate the need for mechanical ventilation [19]. Lopes et al reported that the median need for supplemental oxygen was decreased in the colchicine group (4 vs 6.5 days; *p* < .001) [18]. At day 2, 67% vs 86% of patients maintained the need for supplemental oxygen, while at day 7, the values were 9% vs 42%, in the colchicine and the placebo groups, respectively (log-rank; *p* = .001)

#### 3.4.4. Risk of hospitalisation

The risk of hospitalisation was assessed in the only outpatient study by Tardif et al. [16] The risk was reduced in the colchicine group (4.5% vs 5.7%) but was not statistically significant (*p*-value .08). When groups were subdivided into only PCR positive patients (2075 vs 2084), there was a significant decrease in the hospitalisation risk (4.5% vs 6%; OR 0.75, 95% CI, 0.57–0.99) in the colchicine group.

#### 3.4.5. Length of hospital stay

The results were statistically significant in favour of colchicine in 2 studies. Salehzadeh et al reported a significant decrease (6.28 vs 8.12 days; *p*-value .001) in the length of hospitalisation in the colchicine + HCQ group as compared to the HCQ + placebo group [19]. The median time of hospitalisation was 7.0 vs 9.0 days (*p* = .003) in the colchicine + SOC vs placebo + SOC as reported by Lopes et al. [18] In 2 studies, no statistically significant difference in median hospital duration was observed (12 vs 13 days in Deftereos et al. and 10.5 vs 11 days in Sandhu et al.) [17,22]. Length of hospitalisation was 12.7 vs 11.9 days (aggregate mean) in the study by Mahale et al, however, significance was not reported [20]. Patients discharged on day 28 were approximately five times more in the colchicine group as reported by Burnetti et al (90.9% vs 66.7%: *p*-value .023) [23]. Duration of hospitalisation was not reported in 3 studies.

#### 3.4.6. Adverse effects

Adverse effects were reported by 4 of the 8 studies. Tardif et al reported diarrhoea (13.7% vs. 7.3%; *p* < .001) and pulmonary embolism (PE) (0.5% vs. 0.1%; *p* = .01) in the colchicine group as compared to the placebo group [16]. Deftereos et al. reported diarrhoea in 45.5% vs. 18.0% of patients; *p* = .003 which was self-limited, however lead to drug discontinuation in 1.9% of the patients [17]. Scarsi et al reported that 7.4% of patients had diarrhoea in the colchicine group leading to a decrease in dose to half, no other significant adverse events were reported [21]. New or worsened diarrhoea was more frequent in the intervention group (17% vs 6%; *p*-value .26) and was controlled with the prescription of an antisecretory agent in the study by Lopes et al. [18]

#### 3.4.7. Risk of bias (ROB)

The risk of bias for the RCTs was mixed, with one RCT with high risk, two with some concerns, and one with low risk of bias, with the predicted direction of bias favouring the experimental arm. Overall, the cohort and case-control studies fared poorly in terms of comparability except for the study by Brunetti et al, which had the best quality rating of all the non-RCT studies. Detailed results of the ROB assessment are provided in Table 3, and detailed scoring of individual domains is mentioned in Appendix 2.

**Table 3.** Risk of bias assessment.

Risk of bias for the RCTs as analysed by the Revised Cochrane risk-of-bias tool for randomised trials				
	Salehzadeh et al. [19]	Tardif et al. [16]	Deftereos et al. [17]	Lopes et al. [18]
Randomisation process	High (Favours experimental)	Low	High (Favours experimental)	Low
Deviations from the intended interventions (effect of assignment to intervention)	Low	Low	Low	Some (null)
Deviations from the intended interventions (effect of adhering to intervention)	Low	Low	Low	Low
Missing outcome data	Low	Low	Low	Low
Measurement of the outcome	Low	Low	Low	Low
Selection of the reported result	Some (Favours experimental)	Low	Low	Low
Overall risk of bias	High	Low	Low	Some

Newcastle-Ottawa Scale quality assessment of case-control studies				
	Sandhu et al. [22]	Scarsi et al. [21]		
Selection (max 4 stars)	⋆⋆⋆	⋆⋆⋆⋆		
Comparability (max 2 stars)	–	–		
Exposure (max 3 stars)	⋆⋆	⋆⋆⋆		
Overall (max 9 stars)	⋆⋆⋆⋆⋆	⋆⋆⋆⋆⋆⋆⋆		

Newcastle-Ottawa Scale for quality assessment of cohort studies				
	Mahale et al. [20]	Brunetti et al. [23]		
Selection (max 4 stars)	⋆⋆⋆⋆	⋆⋆⋆⋆		
Comparability (max 2 stars)	–	⋆⋆		
Outcome (max 3 stars)	⋆⋆⋆	⋆⋆⋆		
Overall (max 9 stars)	⋆⋆⋆⋆⋆⋆⋆	⋆⋆⋆⋆⋆⋆⋆⋆⋆		

⋆ Star system for Newcastle-Ottawa Scale (NOS) scores. More stars mean a better rating. Max: maximum. Score of 6 or more for NOS suggestive of higher study quality and credibility

## 4. Discussion

In this systematic review (SR) of 4 RCTs and 4 observational studies, we evaluated the effect of colchicine on mortality, need for mechanical ventilation, change in the inflammatory markers, and adverse effects in COVID-19 patients compared to those who did not receive colchicine. The study designs and clinical outcomes are variable and inconsistent to establish the clinical efficacy of colchicine treatment in COVID-19 for reported outcomes. The certainty of evidence regarding all-cause mortality in the colchicine treatment group was low, as statistically significant low mortality was reported in less than 50% of the studies, and the treatment follow-up period was inconsistently reported. Even the high-quality RCT by Tardif et al. did not show statistically significant benefit on the primary endpoint (death or hospitalisation) in the primary analysis, was underpowered, investigated the clinical outcomes in the outpatient setting, and has the limitation of including patients diagnosed with COVID19 based on clinical criteria which means around 7.3% were not confirmed by PCR [16]. Taken together, the presented data is suggestive of some benefit on patient-related clinical outcomes which need further research. Colchicine is an anti-inflammatory agent that may have a role to prevent hyperinflammatory states as indicated by a reduction in inflammatory markers in patients receiving colchicine in studies evaluating laboratory parameters. Although a decrease in length of stay was reported by 2 studies (Salehzadeh et al and Mahale et al), the presented evidence is of low quality (one study is retrospective, and the RCT is underpowered) [19,20]. This systematic review elucidates that only one study showed a lower rate of intubation in the colchicine group, but this study had many inherited weaknesses with potential performance bias and confounding from additional medications [22]. At this point, it is reasonable to suggest that data is weak and of low quality to suggest a decrease in need for mechanical ventilation with colchicine treatment.

Colchicine has a well-documented safety profile; however, it can cause GI-related side effects (nausea, vomiting, diarrhoea) and has been rarely reported to cause hematological side effects. It may be implied that colchicine's potent anti-inflammatory properties should decrease coagulability, however, on the contrary, the colchicine group had a significantly higher number of PE in the largest study performed by Tardiff et al, whose mechanism is not well studied [16]. It is well documented that COVID-19 is a hypercoagulable state and the anti-inflammatory effects of colchicine could have been negated by the underlying pro-thrombotic state. However, rates of PE were significantly higher in the colchicine group, which is surprising and needs further validation, as earlier studies have not found an increased risk of thromboembolism associated with colchicine. Heterogeneously reported outcomes and variable study designs need further inquiry. Colchicine was used with SOC in most of the studies, but the SOC varied in almost all the studies based on hospital policies and physicians’ discretions. Further, several medications including some antibacterials and antivirals can increase the plasma levels of colchicine by inhibiting CYP3A4 and P-glycoprotein, thus increasing the risk of colchicine adverse effects such as GI-related adverse effects [24]. Inclusion of such medications (e.g. azithromycin) in the SOC by some studies could have enhanced the toxicity reported in those studies. Variable dosing and timing protocols were used for treatment. Adequately powered randomised clinical trials with consistent clinical outcomes, in different clinical settings (hospitalized and out-patient) with a adequate follow-up, are warranted to establish the efficacy of colchicine in COVID-19 treatment. Results from high-quality RCTs are necessary to further prove a treatment benefit of colchicine in COVID-19 patients. The National Institutes of Health and the National Institute for Health and Care Excellence have recommended against the use of colchicine in the management of COVID-19 [25,26].

## 5. Limitations

Our study has several limitations. There were no uniform outcome measures and insufficient reporting of statistical significance of the outcomes which make the precise conclusions challenging. Studies were too variable to perform a meaningful statistical analysis for major outcomes, limiting our study to be a descriptive analysis rather than a meta-analysis. The studies were also heterogeneous in terms of severity of patient population, inclusion criteria, colchicine dosing protocols, comparison group protocols, which makes generalisability and implications of the results unclear. Overall, the quality of evidence was low. Some studies may have been missed as our review was restricted to English language studies.

## 6. Conclusion

Treatment with colchicine in COVID-19 should not be recommended until more evidence is available to support positive outcomes. Based on the available data, judicious and cautious use of colchicine shall be recommended only in clinical trials. Further well-performed clinical trials can assess the efficacy and safety of this drug in COVID-19.

## Supplementary Material

Supplemental Material

## Data Availability

The authors confirm that the data supporting the findings of this study are available within the article and its supplementary materials.

## Appendix. Impact of colchicine on mortality and morbidity in COVID-19: a systematic review

**Search strategy**

Database: Embase <1988 to 2021 Week 05>

––––––––––––––––––––––––––––––––––––––––

1 covid.mp. (85089)

2 covid-19.mp. (84109)

3 SARS-CoV-2.mp. (30120)

4 exp Coronavirinae/(22598)

5 severe acute respiratory syndrome coronavirus 2.mp. (26678)

6 sars cov 2.mp. (30120)

7 ncov.mp. (1684)

8 2019 ncov.mp. (1506)

9 1 or 2 or 3 or 4 or 5 or 6 or 7 or 8 (104719)

10 colchicine/(24785)

11 Colcrys.mp. (73)

12 mitigare.mp. (2)

13 10 or 11 or 12 (24787)

14 9 and 13 (201)

15 limit 14 to english language (199)

***************************

**Table:**

Database	# of initial citations
Embase	199
PubMed	95
medRxiv	46
Scopus	160
Prospero	11
Google Scholar	210
Additional records from other sources	1
	
Total citations	721
Duplicates	323
End result	399

## Appendix 2. Impact of colchicine on mortality and morbidity in COVID-19: a systematic review

Risk of bias for the RCTs as analysed by the Revised Cochrane risk-of-bias tool for randomised trials

**Table:**

Domain 1: Risk of bias arising from the randomisation process			Salehzadeh et al.	Tardif et al.	Deftereos et al.	Lopes et al.
Signalling question	Comment	Response options	Response	Response	Response	Response
1.1. Was the allocation sequence random?		Y/PY/PN/N/NI	NI	PY	Y	Y
1.2. Was the allocation sequence concealed until participants were enrolled and assigned to interventions?		Y/PY/PN/N/NI	NI	Y	N	Y
1.3. Did baseline differences between intervention groups suggest a problem with the randomisation process?		Y/PY/PN/N/NI	PY	N	N	N
Risk-of-bias judgement		Low/High/Some concerns	High	Low	High	Low
What is the predicted direction of bias arising from the randomisation process?		NA/Favours experimental/Favours comparator/Towards null /Away from null/Unpredictable	Favours Experimental		Favours experimental	
						
Domain 2: Risk of bias due to deviations from the intended interventions (effect of assignment to intervention)			Salehzadeh et al.	Tardif et al.	Deftereos et al.	Lopes et al.
Signalling question	Comment	Response options	Response	Response	Response	Response
2.1. Were participants aware of their assigned intervention during the trial?		Y/PY/PN/N/NI	N	N	Y	N
2.2. Were carers and people delivering the interventions aware of participants' assigned intervention during the trial?		Y/PY/PN/N/NI	PN	N	Y	PN
2.3. If Y/PY/NI to 2.1 or 2.2. Were there deviations from the intended intervention that arose because of the trial context?		Y/PY/PN/N/NI			N	
2.4. If Y/PY to 2.3: Were these deviations likely to have affected the outcome?		Y/PY/PN/N/NI				
2.5. If Y/PY/NI to 2.4: Were these deviations from intended intervention balanced between groups?		Y/PY/PN/N/NI				
2.6. Was an appropriate analysis used to estimate the effect of assignment to intervention?		Y/PY/PN/N/NI	PY	Y	Y	PN
2.7. If N/PN/NI to 2.6: Was there potential for a substantial impact (on the result) of the failure to analyse participants in the group to which they were randomised?		Y/PY/PN/N/NI				PN
Risk-of-bias judgement		Low/High/Some concerns	Low	Low	Low	Some
What is the predicted direction of bias arising from the randomisation process?		NA/Favours experimental/Favours comparator/Towards null /Away from null/Unpredictable				Null
						
Domain 2: Risk of bias due to deviations from the intended interventions (effect of adhering to intervention)			Salehzadeh et al.	Tardif et al.	Deftereos et al.	Lopes et al.
Signalling question	Comment	Response options	Response	Response	Response	Response
2.1. Were participants aware of their assigned intervention during the trial?		Y/PY/PN/N/NI	N	N	Y	N
2.2. Were carers and people delivering the interventions aware of participants' assigned intervention during the trial?		Y/PY/PN/N/NI	N	N	Y	N
2.3. [If applicable:] If Y/PY/NI to 2.1 or 2.2: Were important non-protocol interventions balanced across intervention groups?		Y/PY/PN/N/NI			PY	
2.4. [If applicable:] Were there failures in implementing the intervention that could have affected the outcome?		Y/PY/PN/N/NI	N	N	N	N
2.5. [If applicable:] Was there non-adherence to the assigned intervention regimen that could have affected participants’ outcomes?		Y/PY/PN/N/NI	N	PN	N	N
2.6. If N/PN/NI to 2.3, or Y/PY/NI to 2.4 or 2.5: Was an appropriate analysis used to estimate the effect of adhering to the intervention?		Y/PY/PN/N/NI				
Risk-of-bias judgement		Low/High/Some concerns	Low	Low	Low	Low
What is the predicted direction of bias arising from the randomisation process?		NA/Favours experimental/Favours comparator/Towards null /Away from null/Unpredictable				
						
Domain 3: Risk of bias due to missing outcome data			Salehzadeh et al.	Tardif et al.	Deftereos et al.	Lopes et al.
Signalling question	Comment	Response options	Response	Response	Response	Response
3.1. Were data for this outcome available for all, or nearly all, participants randomised?		Y/PY/PN/N/NI	Y	Y	Y	Y
3.2. If N/PN/NI to 3.1: Is there evidence that the result was not biased by missing outcome data?		Y/PY/PN/N/NI				
3.3. If N/PN to 3.2: Could missingness in the outcome depend on its true value?		Y/PY/PN/N/NI				
3.4. If Y/PY/NI to 3.3: Is it likely that missingness in the outcome depended on its true value?		Y/PY/PN/N/NI				
Risk-of-bias judgement		Low/High/Some concerns	Low	Low	Low	Low
What is the predicted direction of bias arising from the randomisation process?		NA/Favours experimental/Favours comparator/Towards null /Away from null/Unpredictable				
						
Domain 4: Risk of bias in measurement of the outcome			Salehzadeh et al.	Tardif et al.	Deftereos et al.	Lopes et al.
Signalling question	Comment	Response options	Response	Response	Response	Response
4.1. Was the method of measuring the outcome inappropriate?		Y/PY/PN/N/NI	PN	PN	PN	PN
4.2. Could measurement or ascertainment of the outcome have differed between intervention groups?		Y/PY/PN/N/NI	PN	PN	PN	PN
4.3. If N/PN/NI to 4.1 and 4.2: Were outcome assessors aware of the intervention received by study participants?		Y/PY/PN/N/NI	PN	PN	Y	PN
4.4. If Y/PY/NI to 4.3: Could assessment of the outcome have been influenced by knowledge of intervention received?		Y/PY/PN/N/NI			PN	
4.5. If Y/PY/NI to 4.4: Is it likely that assessment of the outcome was influenced by knowledge of intervention received?		Y/PY/PN/N/NI				
Risk-of-bias judgement		Low/High/Some concerns	Low	Low	Low	Low
What is the predicted direction of bias arising from the randomisation process?		NA/Favours experimental/Favours comparator/Towards null /Away from null/Unpredictable				
						
Domain 5: Risk of bias in selection of the reported result			Salehzadeh et al.	Tardif et al.	Deftereos et al.	Lopes et al.
Signalling question	Comment	Response options	Response	Response	Response	Response
5.1. Were the data that produced this result analysed in accordance with a pre-specified analysis plan that was finalised before unblinded outcome data were available for analysis?		Y/PY/PN/N/NI	NI	PY	PY	PY
5.2. Is the numerical result being assessed likely to have been selected, on the basis of the results, from multiple eligible outcome measurements (e.g. scales, definitions, time points) within theoutcome domain?		Y/PY/PN/N/NI	NI	PN	PN	PN
5.2. Is the numerical result being assessed likely to have been selected, on the basis of the results, from multiple eligible analyses of the data?		Y/PY/PN/N/NI	NI	PN	PN	PN
Risk-of-bias judgement		Low/High/Some concerns	Some	Low	Low	Low
What is the predicted direction of bias arising from the randomisation process?		NA/Favours experimental/Favours comparator/Towards null /Away from null/Unpredictable	Favours experimental			
						
			Salehzadeh et al.	Tardif et al.	Deftereos et al.	Lopes et al.
Overall risk-of-bias judgement		Low risk of bias/Some concerns/High risk of bias	High	Low	Low	Some

**Table:** Newcastle-Ottawa Scale quality assessment of case-control studies

		Sandhu et al.	Scarsi et al.
Selection			
Is the case definition accurate	1 star if Yes with independent vaidation	*	*
Representativeness of cases	1 star if consecutive or obviously representative series of cases	*	*
Selection of controls	1 star community controls	---	*
Definition of controls	1 star if no history of exposure or endpoints	*	*
			
Comparability			
Comparability of cohorts on the basis of the design or analysis	1 star if study controls for (most important factor – severity of COVID illness)	----	----
Comparability of cohorts on the basis of the design or analysis	1 star if study controls for (2nd most important factor – comorbidities)	----	----
			
Exposure			
Ascertainment of exposure	1 star if secure record or structured interview where blind to case/control status	*	*
Same method of ascertainment for cases and controls	1 star if Yes	*	*
Non-response rate	1 star if same rate for both groups	----	*
			
Total stars		5	7
