# Supplementary material for: Impact of colchicine on mortality and morbidity in COVID-19: a systematic review
Source: Ann Med. 2022 Mar 8;54(1):775–89. doi: 10.1080/07853890.2021.1993327 (PMC8920395; doi:10.1080/07853890.2021.1993327)
Supplement: Supplemental Material [file IANN_A_1993327_SM8231.docx]

**Impact of Colchicine on Mortality and Morbidity in COVID-19: A Systematic Review: Appendix.**

**Search Strategy**

Database: Embase <1988 to 2021 Week 05>

--------------------------------------------------------------------------------

1 covid.mp. (85089)

2 covid-19.mp. (84109)

3 SARS-CoV-2.mp. (30120)

4 exp Coronavirinae/ (22598)

5 severe acute respiratory syndrome coronavirus 2.mp. (26678)

6 sars cov 2.mp. (30120)

7 ncov.mp. (1684)

8 2019 ncov.mp. (1506)

9 1 or 2 or 3 or 4 or 5 or 6 or 7 or 8 (104719)

10 colchicine/ (24785)

11 Colcrys.mp. (73)

12 mitigare.mp. (2)

13 10 or 11 or 12 (24787)

14 9 and 13 (201)

15 limit 14 to english language (199)

***************************

| **Database** | **# of initial citations** |
| --- | --- |
| Embase | 199 |
| PubMed | 95 |
| medRxiv | 46 |
| Scopus | 160 |
| Prospero | 11 |
| Google Scholar | 210 |
| Additional records from other sources | 1 |
|  |  |
| **Total Citations** | 721 |
| Duplicates | 323 |
| **End Result** | **399** |

**Impact of Colchicine on Mortality and Morbidity in COVID-19: A Systematic Review: Appendix.**

**Risk of bias for the RCTs as analyzed by the Revised Cochrane risk-of-bias tool for randomized trials**

| **Domain 1: Risk of bias arising from the randomization process** | | | | | **Salehzadeh et al.** | | **Tardif et al.** | | **Deftereos et al.** | **Lopes et al.** |  |
| --- | --- | --- | --- | --- | --- | --- | --- | --- | --- | --- | --- |
| **Signalling question** | | **Comment** | **Response options** | | **Response** | | **Response** | | **Response** | **Response** |  |
| 1.1 Was the allocation sequence random? | |  | Y/PY/PN/N/NI | | NI | | PY | | Y | Y |  |
| 1.2 Was the allocation sequence concealed until participants were enrolled and assigned to interventions? | |  | Y/PY/PN/N/NI | | NI | | Y | | N | Y |  |
| 1.3 Did baseline differences between intervention groups suggest a problem with the randomization process? | |  | Y/PY/PN/N/NI | | PY | | N | | N | N |  |
| **Risk-of-bias judgement** | |  | Low / High / Some concerns | | High | | Low | | High | Low |  |
| What is the predicted direction of bias arising from the randomization process? | |  | NA / Favours experimental / Favours comparator / Towards null /Away from null / Unpredictable | | Favours Experimental | |  | | Favours experimental |  |  |
|  | |  |  | |  | |  | |  |  |  |
| **Domain 2: Risk of bias due to deviations from the intended interventions (effect of assignment to intervention)** | | | | | **Salehzadeh et al.** | | **Tardif et al.** | | **Deftereos et al.** | **Lopes et al.** |  |
| **Signalling question** | | **Comment** | **Response options** | | **Response** | | **Response** | | **Response** | **Response** |  |
| 2.1. Were participants aware of their assigned intervention during the trial? | |  | Y/PY/PN/N/NI | | N | | N | | Y | N |  |
| 2.2. Were carers and people delivering the interventions aware of participants' assigned intervention during the trial? | |  | Y/PY/PN/N/NI | | PN | | N | | Y | PN |  |
| 2.3. If Y/PY/NI to 2.1 or 2.2: Were there deviations from the intended intervention that arose because of the trial context? | |  | Y/PY/PN/N/NI | |  | |  | | N |  |  |
| 2.4 If Y/PY to 2.3: Were these deviations likely to have affected the outcome? | |  | Y/PY/PN/N/NI | |  | |  | |  |  |  |
| 2.5. If Y/PY/NI to 2.4: Were these deviations from intended intervention balanced between groups? | |  | Y/PY/PN/N/NI | |  | |  | |  |  |  |
| 2.6 Was an appropriate analysis used to estimate the effect of assignment to intervention? | |  | Y/PY/PN/N/NI | | PY | | Y | | Y | PN |  |
| 2.7 If N/PN/NI to 2.6: Was there potential for a substantial impact (on the result) of the failure to analyse participants in the group to which they were randomized? | |  | Y/PY/PN/N/NI | |  | |  | |  | PN |  |
| **Risk-of-bias judgement** | |  | Low / High / Some concerns | | Low | | Low | | Low | Some |  |
| What is the predicted direction of bias arising from the randomization process? | |  | NA / Favours experimental / Favours comparator / Towards null /Away from null / Unpredictable | |  | |  | |  | Null |  |
|  | |  |  | |  | |  | |  |  |  |
| **Domain 2: Risk of bias due to deviations from the intended interventions (effect of adhering to intervention)** | | | | | **Salehzadeh et al.** | | **Tardif et al.** | | **Deftereos et al.** | **Lopes et al.** |  |
| **Signalling question** | | **Comment** | **Response options** | | **Response** | | **Response** | | **Response** | **Response** |  |
| 2.1. Were participants aware of their assigned intervention during the trial? | |  | Y/PY/PN/N/NI | | N | | N | | Y | N |  |
| 2.2. Were carers and people delivering the interventions aware of participants' assigned intervention during the trial? | |  | Y/PY/PN/N/NI | | N | | N | | Y | N |  |
| 2.3. [If applicable:] If Y/PY/NI to 2.1 or 2.2: Were important non-protocol interventions balanced across intervention groups? | |  | Y/PY/PN/N/NI | |  | |  | | PY |  |  |
| 2.4. [If applicable:] Were there failures in implementing the intervention that could have affected the outcome? | |  | Y/PY/PN/N/NI | | N | | N | | N | N |  |
| 2.5. [If applicable:] Was there non-adherence to the assigned intervention regimen that could have affected participants’ outcomes? | |  | Y/PY/PN/N/NI | | N | | PN | | N | N |  |
| 2.6. If N/PN/NI to 2.3, or Y/PY/NI to 2.4 or 2.5: Was an appropriate analysis used to estimate the effect of adhering to the intervention? | |  | Y/PY/PN/N/NI | |  | |  | |  |  |  |
| **Risk-of-bias judgement** | |  | Low / High / Some concerns | | Low | | Low | | Low | Low |  |
| What is the predicted direction of bias arising from the randomization process? | |  | NA / Favours experimental / Favours comparator / Towards null /Away from null / Unpredictable | |  | |  | |  |  |  |
|  | |  |  | |  | |  | |  |  |  |
| **Domain 3: Risk of bias due to missing outcome data** | | | | | **Salehzadeh et al.** | | **Tardif et al.** | | **Deftereos et al.** | **Lopes et al.** |  |
| **Signalling question** | | **Comment** | **Response options** | | **Response** | | **Response** | | **Response** | **Response** |  |
| 3.1 Were data for this outcome available for all, or nearly all, participants randomized? | |  | Y/PY/PN/N/NI | | Y | | Y | | Y | Y |  |
| 3.2 If N/PN/NI to 3.1: Is there evidence that the result was not biased by missing outcome data? | |  | Y/PY/PN/N/NI | |  | |  | |  |  |  |
| 3.3 If N/PN to 3.2: Could missingness in the outcome depend on its true value? | |  | Y/PY/PN/N/NI | |  | |  | |  |  |  |
| 3.4 If Y/PY/NI to 3.3: Is it likely that missingness in the outcome depended on its true value? | |  | Y/PY/PN/N/NI | |  | |  | |  |  |  |
| **Risk-of-bias judgement** | |  | Low / High / Some concerns | | Low | | Low | | Low | Low |  |
| What is the predicted direction of bias arising from the randomization process? | |  | NA / Favours experimental / Favours comparator / Towards null /Away from null / Unpredictable | |  | |  | |  |  |  |
|  | |  |  | |  | |  | |  |  |  |
| **Domain 4: Risk of bias in measurement of the outcome** | | | | | **Salehzadeh et al.** | | **Tardif et al.** | | **Deftereos et al.** | **Lopes et al.** |  |
| **Signalling question** | | **Comment** | **Response options** | | **Response** | | **Response** | | **Response** | **Response** |  |
| 4.1 Was the method of measuring the outcome inappropriate? | |  | Y/PY/PN/N/NI | | PN | | PN | | PN | PN |  |
| 4.2 Could measurement or ascertainment of the outcome have differed between intervention groups? | |  | Y/PY/PN/N/NI | | PN | | PN | | PN | PN |  |
| 4.3 If N/PN/NI to 4.1 and 4.2: Were outcome assessors aware of the intervention received by study participants? | |  | Y/PY/PN/N/NI | | PN | | PN | | Y | PN |  |
| 4.4 If Y/PY/NI to 4.3: Could assessment of the outcome have been influenced by knowledge of intervention received? | |  | Y/PY/PN/N/NI | |  | |  | | PN |  |  |
| 4.5 If Y/PY/NI to 4.4: Is it likely that assessment of the outcome was influenced by knowledge of intervention received? | |  | Y/PY/PN/N/NI | |  | |  | |  |  |  |
| **Risk-of-bias judgement** | |  | Low / High / Some concerns | | Low | | Low | | Low | Low |  |
| What is the predicted direction of bias arising from the randomization process? | |  | NA / Favours experimental / Favours comparator / Towards null /Away from null / Unpredictable | |  | |  | |  |  |  |
|  | |  |  | |  | |  | |  |  |  |
| **Domain 5: Risk of bias in selection of the reported result** | | | | | **Salehzadeh et al.** | | **Tardif et al.** | | **Deftereos et al.** | **Lopes et al.** |  |
| **Signalling question** | | **Comment** | **Response options** | | **Response** | | **Response** | | **Response** | **Response** |  |
| 5.1 Were the data that produced this result analysed in accordance with a pre-specified analysis plan that was finalized before unblinded outcome data were available for analysis? | |  | Y/PY/PN/N/NI | | NI | | PY | | PY | PY |  |
| 5.2 Is the numerical result being assessed likely to have been selected, on the basis of the results, from multiple eligible outcome measurements (e.g. scales, definitions, time points) within theoutcome domain? | |  | Y/PY/PN/N/NI | | NI | | PN | | PN | PN |  |
| 5.2 Is the numerical result being assessed likely to have been selected, on the basis of the results, from multiple eligible analyses of the data? | |  | Y/PY/PN/N/NI | | NI | | PN | | PN | PN |  |
| **Risk-of-bias judgement** | |  | Low / High / Some concerns | | Some | | Low | | Low | Low |  |
| What is the predicted direction of bias arising from the randomization process? | |  | NA / Favours experimental / Favours comparator / Towards null /Away from null / Unpredictable | | Favours experimental | |  | |  |  |  |
|  | |  |  | |  | |  | |  |  |  |
|  | |  |  | | **Salehzadeh et al.** | | **Tardif et al.** | | **Deftereos et al.** | **Lopes et al.** |  |
| **Overall risk-of-bias judgement** | |  | **Low risk of bias / Some concerns / High risk of bias** | | **High** | | **Low** | | **Low** | **Some** |  |
| **Newcastle-Ottawa Scale quality assessment of case-control studies** | | | | | | | | | | | |
|  |  | | | **Mahale et al.** | | **Brunetti et al.** | |  |  |  |  |
| **SELECTION** | | | |  | |  | |  |  |  |  |
| Representativeness of the exposed cohort | 1 star if truly or somewhat representative of the average | | | * | | * | |  |  |  |  |
| Selection of non-exposed cohort | 1 star if drawn from the same community as the exposed cohort | | | * | | * | |  |  |  |  |
| Ascertainment of exposure | 1 star if secure record or structured interview | | | * | | * | |  |  |  |  |
| Demonstration that outcome of interest was not present at start of study | 1 star if Yes | | | * | | * | |  |  |  |  |
|  |  | | |  | |  | |  |  |  |  |
| **COMPARABILITY** | | | |  | |  | |  |  |  |  |
| Comparability of cohorts on the basis of the design or analysis | 1 star if study controls for (most important factor - severity of COVID illness) | | | ---- | | * | |  |  |  |  |
| Comparability of cohorts on the basis of the design or analysis | 1 star if study controls for (2nd most important factor - comorbidities) | | | ---- | | * | |  |  |  |  |
|  |  | | |  | |  | |  |  |  |  |
| **OUTCOME** | | | |  | |  | |  |  |  |  |
| Assessment of outcome | 1 star if independent blind assessment or record linkage | | | * | | * | |  |  |  |  |
| Was follow-up long enough for outcomes to occur | 1 star if Yes | | | * | | * | |  |  |  |  |
| Adequacy of follow up of cohorts | 1 star if complete follow up - all subjects accounted for. OR 1 star if subjects lost to follow up unlikely to introduce bias - small number lost (follow up rate > ___% OR description provided for those lost) | | | * | | * | |  |  |  |  |
|  |  | | |  | |  | |  |  |  |  |
| **TOTAL STARS** | | | | 7 | | 9 | |  |  |  |  |

**Newcastle-Ottawa Scale for quality assessment of cohort studies**

|  |  | **Sandhu et al.** | **Scarsi et al.** |
| --- | --- | --- | --- |
| **SELECTION** | |  |  |
| Is the case definition accurate | 1 star if Yes with independent vaidation | * | * |
| Representativeness of cases | 1 star if consecutive or obviously representative series of cases | * | * |
| Selection of controls | 1 star community controls | --- | * |
| Definition of controls | 1 star if no history of exposure or endpoints | * | * |
|  |  |  |  |
| **COMPARABILITY** | |  |  |
| Comparability of cohorts on the basis of the design or analysis | 1 star if study controls for (most important factor - severity of COVID illness) | ---- | ---- |
| Comparability of cohorts on the basis of the design or analysis | 1 star if study controls for (2nd most important factor - comorbidities) | ---- | ---- |
|  |  |  |  |
| **EXPOSURE** | |  |  |
| Ascertainment of exposure | 1 star if secure record or structured interview where blind to case/control status | * | * |
| Same method of ascertainment for cases and controls | 1 star if Yes | * | * |
| Non-response rate | 1 star if same rate for both groups | ---- | * |
|  |  |  |  |
| **TOTAL STARS** | | 5 | 7 |
